# Editorial: Immunotherapeutics development against Hantaviruses

**DOI:** 10.3389/fimmu.2024.1377137

**Published:** 2024-02-07

**Authors:** Muhammad Tahir ul Qamar, Sajjad Ahmad, Abbas Khan, Dongqing Wei

**Affiliations:** ^1^ Integrative Omics and Molecular Modeling Laboratory, Department of Bioinformatics and Biotechnology, Government College University Faisalabad (GCUF), Faisalabad, Pakistan; ^2^ Department of Health and Biological Sciences, Abasyn University, Peshawar, Pakistan; ^3^ Gilbert and Rose-Marie Chagoury School of Medicine, Lebanese American University, Beirut, Lebanon; ^4^ Department of Natural Sciences, Lebanese American University, Beirut, Lebanon; ^5^ Department of Bioinformatics and Biostatistics, School of Life Sciences and Biotechnology, Shanghai Jiao Tong University, Shanghai, China

**Keywords:** hantaviruses, computational biology, RNA-based therapeutics, vaccine development, antiviral drugs

## 1 Introduction

In the realm of infectious diseases, Hantaviruses represent a significant global health challenge, characterized by their negative single-stranded RNA (ssRNA) structure and classification within the Hantaviridae family and Bunyavirales order. Annually, these viruses are responsible for infecting approximately 150,000 to 200,000 individuals worldwide, with 40 identified species, 22 of which pose a greater risk of contagious infections. Despite the widespread impact of these viruses, the medical community faces a notable gap in FDA-approved treatments, underscoring an urgent need for advanced research and development in immunotherapeutics. This editorial endeavors to shed light on innovative strides within this domain, focusing on novel vaccine and drug development strategies, omics data housing, RNA-based therapeutics, and computational biology insights aimed at comprehensively understanding and managing Hantavirus biology. The culmination of efforts in these areas is pivotal not only for advancing our scientific understanding but also for paving the way toward effective treatments.

## 2 Advancements in Hantavirus immunotherapeutics: a closer look at pioneering studies

In this editorial, we discuss the five significant studies that represent the forefront of these efforts, showcasing innovative approaches in vaccine and drug development, as well as advancements in diagnostic technologies.

A study led by Noor et al. provided a comprehensive computational analysis of viral motifs, particularly focusing on the YXXΦ[I/L/M/F/V] motif and YXXΦ-like tetrapeptides in HFRS-causing Hantaviruses. By elucidating the association between these motifs and viral pathogenesis, as well as their role in host immune regulation, the research offers new insights into potential targets for therapeutic intervention.

Another research conducted by Alshammari explored the identification of new inhibitors against Hantaviruses using advanced molecular modeling techniques. This approach underscores the importance of molecular dynamics and chemistry in discovering compounds with the potential to disrupt viral replication and assembly.

The work of Alabbas employed an innovative combination of subtractive proteomics, immunoinformatics, and molecular simulation to identify promising candidates for vaccines. This integrated approach highlights the significant potential of bioinformatics and computational biology to speed up the vaccine development process by identifying antigens that can trigger strong immune responses.

Similarly, another significant contribution from Noor et al. investigated the codon usage patterns in HFRS-causing Hantaviruses. This study provides insights into the evolutionary dynamics of these viruses and their adaptation strategies, offering valuable information for the development of effective vaccines and therapeutics.

Wang and Wei provided an updated review on the contemporary methodologies for RNA virus detection. Their analysis elucidates a state-of-the-art approach, particularly highlighting the innovative integration of nucleic acid testing with immunoassays via single-molecule digital ELISA.

## 3 Scientific and global health implications: enhancing the fight against Hantaviruses

The contributions of these studies are manifold, offering not only a deeper understanding of Hantavirus biology and pathogenesis but also paving the way for innovative therapeutic and diagnostic solutions. The interdisciplinary nature of the research underscores the necessity of combining computational, molecular, and immunological approaches to tackle the complexities of viral diseases. Further, these scientific advancements have profound implications for global health infrastructure and policy. The development of effective therapeutics and vaccines against Hantaviruses has the potential to significantly reduce disease burden, particularly in endemic regions. Additionally, the improvement in diagnostic capabilities ensures better surveillance and outbreak management, critical components of global health security.

## 4 Navigating challenges and setting future directions

Despite these advancements, the journey toward eradicating Hantavirus-related diseases is fraught with obstacles. One of the primary challenges lies in the genetic diversity and adaptability of Hantaviruses, which complicates the development of universal vaccines and therapeutics. Furthermore, the varying severity of disease manifestations and the complex interactions between the viruses and their hosts necessitate a deeper understanding and more sophisticated approaches to treatment and prevention. Looking ahead, the path forward must include enhanced global surveillance systems to monitor Hantavirus infections and potential outbreaks. Thus, continued investment in research and development is critical, with a focus on interdisciplinary collaborations that leverage the strengths of computational biology, molecular genetics, immunology, and epidemiology.

## 5 Conclusion

The Research Topic dedicated to the development of immunotherapeutics against Hantaviruses highlights significant scientific achievements and sets the stage for future research and public health initiatives. The studies discussed herein not only contribute to our understanding of Hantavirus pathogenesis but also pave the way for innovative treatments and diagnostic tools. As we navigate the challenges ahead, it is imperative that the global scientific and health communities come together to continue this momentum, ensuring that the fight against Hantaviruses and other infectious diseases remains a top priority. Through collaborative efforts and a commitment to innovation, we can look forward to a future where the impact of these viruses is significantly diminished.

## Author contributions

MQ: Writing – original draft, Writing – review & editing. SA: Writing – original draft, Writing – review & editing. AK: Writing – original draft, Writing – review & editing. DW: Writing – original draft, Writing – review & editing.

